# MotifAdjuster: a tool for computational reassessment of transcription factor binding site annotations

**DOI:** 10.1186/gb-2009-10-5-r46

**Published:** 2009-05-01

**Authors:** Jens Keilwagen, Jan Baumbach, Thomas A Kohl, Ivo Grosse

**Affiliations:** 1 Leibniz Institute of Plant Genetics and Crop Plant Research Gatersleben (IPK), Corrensstraße 3, 06466 Gatersleben, Germany; 2 International Computer Science Institute, 1947 Center Street, Berkeley, California 94704, USA; 3 International NRW Graduate School in Bioinformatics and Genome Research, Center for Biotechnology (CeBiTec), Bielefeld University, Universitätsstraße 27, 33615 Bielefeld, Germany; 4 Institute for Genome Research and Systems Biology (IGS), Center for Biotechnology (CeBiTec), Bielefeld University, Universitätsstraße 27, 33615 Bielefeld, Germany; 5 Institute of Computer Science, Martin Luther University Halle-Wittenberg, Von-Seckendorff-Platz 1, 06120 Halle, Germany

## Abstract

Valuable binding-site annotation data are stored in databases. However, several types of errors can, and do, occur in the process of manually incorporating annotation data from the scientific literature into these databases. Here, we introduce MotifAdjuster , a tool that helps to detect these errors, and we demonstrate its efficacy on public data sets.

## Abstract 2

MotifAdjuster helps to detect errors in binding site annotations.

## Rationale

The regulation of gene expression involves a complex system of interacting components in all living organisms [1] and is of fundamental interest, for instance, for cell maintenance and development. One level of regulation is realized by DNA-binding transcription factors (TFs). The DNA-binding domain of a TF is capable of recognizing specific binding sites (BSs) in the promoter regions of its target genes [2]. Binding of a TF can induce (activator) or inhibit (repressor) the transcription of its target genes. The general ability to control a target gene may depend on the BS itself, its strand orientation, and its position with respect to the transcription start site. If other BSs are present, the ability of a TF to bind the DNA may additionally depend on strand orientations and positions of these BSs.

One important prerequisite for research on gene regulation is the reliable annotation of BSs. The approximate regions on the double-stranded DNA sequence bound by TFs can be determined by wet-lab experiments such as electrophoretic mobility shift assays (EMSAs) [3], DNAse footprinting [4], enzyme-linked immunosorbent assay (ELISA) [5,6], ChIP-chip [7], or mutations of the putative BS and subsequent expression studies. Because TFs bind to double-stranded DNA, the strand annotations of nonpalindromic BSs in the databases are either missing or added, based on manual inspection or predictions from bioinformatics tools such as MEME [8], Gibbs Sampler [9,10], Improbizer [11], SeSiMCMC [12], or A-GLAM [13].

After wet-lab identification, data about transcriptional gene regulatory interactions, including the annotated BSs, are published in the scientific literature. Subsequently, these data are extracted by curation teams and manually entered into databases on transcriptional gene regulation such as CoryneRegNet [14], PRODORIC [15], or RegulonDB [16] for prokaryotes, and AGRIS [17], AthaMap [18], CTCFBSDB [19], JASPAR [20], OregAnno [21], SCPD [22], TRANSFAC [23], TRED [24], or TRRD [25] for eukaryotes. Three typical problems may occur during the process of transferring these data.

First, erroneously annotated BS: This error may occur in the original study or during the transfer process from the scientific literature to the databases. A sequence is declared to contain a BS, although, in reality, it does not.

Second, shift of the BS: The BS may be erroneously shifted by one or a few base pairs. This typically happens during the transfer process from the scientific literature to the databases.

Third, missing or wrong strand orientation of the BS: The strand orientation of a BS is often not or incorrectly annotated. For example, all BS orientations are arbitrarily declared to be in 5'→3' direction relative to the target gene in CoryneRegNet and in RegulonDB [14,16].

These problems can strongly affect any of the subsequent analysis steps, such as the inference of sequence motifs from "experimentally verified" data, the calculation of *P *values for the occurrence of BSs, the detection of putative BSs in genome-wide scans and their experimental validation, or the reconstruction of transcriptional gene-regulatory networks.

Here, we introduce MotifAdjuster, a software tool for detecting potential BS annotation errors and for proposing possible corrections. Existing bioinformatics tools [8-13] are not optimized for this task (Additional data file 1), because they do not allow shifting the BS by using a nonuniform distribution and considering both strands with unequal weights. In contrast, MotifAdjuster allows the user to incorporate prior knowledge about (i) the probability of erroneously annotated BSs, (ii) the distribution of possible shifts, and (iii) the strand preference.

One widely-used model for the representation of BSs is the position weight matrix (PWM) model [8-13,26,27], and many software tools for genome-wide scans of sequence motifs are based on PWM models [26,28,29]. MotifAdjuster is based on a simple mixture model using a PWM model on both strands for the motif sequences and a homogeneous Markov model of order 0 for the flanking sequences similar to MEME, Gibbs Sampler, Improbizer, SeSiMCMC, or A-GLAM. For a given set of BSs, MotifAdjuster tests whether each sequence contains a BS, and it refines the annotations of position and strand for each BS, if necessary, by maximizing the posterior of the mixture model by using a simple *expectation maximization *(EM) algorithm.

To test the efficacy of MotifAdjuster, we apply it to seven data sets from CoryneRegNet, and we record for each of them the set of potential annotation errors. For one example, the nitrate regulator NarL, we compare the proposed adjustments with the original literature, with a manual strand reannotation of the BS strands, and with an independent and hand-curated reannotation provided by PRODORIC. Finally, we test whether the PWM estimated from the adjusted NarL BSs can help to detect unknown BSs in those promoter regions that are known to be bound by NarL, but for which no BS could be predicted in the past.

## Algorithm

In this section, we present the MotifAdjuster algorithm including the mixture model, the prior, and the maximum *a posteriori *(MAP) estimation of the model parameters given the data.

### Mixture model

We denote a DNA sequence of length *L *by *x*:= (*x*_1_, *x*_2_, ..., *x*_*L*_), the nucleotide at position *ℓ *∈ [1, *L*] by *x*_*ℓ *_∈ {*A*, *C*, *G*, *T*}, and the *reverse complement *of *x* by *x*^*RC*^. For modeling a BS *x* of length *w*, we use a PWM model, which assumes that the nucleotides at all positions are statistically independent of each other, resulting in an additive log-likelihood

(1)

of sequence *x* given the model parameters *λ* [30,31], where the subscript *f *stands for *foreground*. Here,  denotes the logarithm of the probability of finding nucleotide *a *∈ {*A*, *C*, *G*, *T*} at position *ℓ*, *λ*^*ℓ *^denotes the four-dimensional vector , and *λ* denotes the (4 × *w*) matrix, that is, *λ* denotes the PWM [32-36].

For modeling the flanking sequences, we use a homogeneous Markov model of order 0, which assumes that all nucleotides are statistically independent, resulting in an additive log-likelihood

(2)

of sequence *x* given model parameters *τ* [32-36], where the subscript *b *stands for *background*. Here, *τ*_*a *_denotes the logarithm of the probability of nucleotide *a*, and *τ* denotes the vector (*τ*_*A*_, ..., *τ*_*T*_)^*T*^.

For the detection of sequences (i) erroneously annotated as containing BSs, (ii) with shifted BSs, or (iii) with missing or wrong strand annotations, we introduce the three random variables *u*_1_, *u*_2_, and *u*_3_.

The variable *u*_1 _handles the possibility that a sequence annotated as containing a BS does not contain a BS. *u*_1 _= 0 denotes the case that the sequence contains no BS, and *u*_1 _= 1 denotes the case that the sequence contains exactly one BS. If the sequence contains one BS, it can be located at different positions and on both strands.

The variable *u*_2 _handles the possibility of shifts of a BS caused by annotation errors. *u*_2 _models the start position of the BS in the sequence with respect to the annotated start position. This variable can assume the integer values {-*s*, -(*s*-1), ..., *s*-1, *s*}, where *s *is the maximal shift of the BS upstream or downstream of the annotated position.

The variable *u*_3 _handles the possibility that a BS can have two orientations in the double-stranded upstream region of the target gene. According to the notation of CoryneRegNet, *u*_3 _= 0 denotes the forward strand defined as the strand in 5'→3' direction relative to the target gene, and *u*_3 _= 1 denotes the reverse complementary strand.

For shortness of notation, we define *u* := (*u*_1_, *u*_2_, *u*_3_). Because we do not know the values of *u*, these variables are modeled as hidden variables. We assume that *u*_2 _and *u*_3 _are conditionally independent of each other given *u*_1_; that is, we assume that annotation errors of position and strand are conditionally independent given the occurrence of the BS. We define

(3)

where the subscript *h *stands for *hidden*, and where *ɸ *:= (*ɸ*_1_, *ɸ*_2_, *ɸ*_3_) denotes the vector of parameters of this distribution. MotifAdjuster allows the user to specify the probability *P*_*h *_(*u*_1_|*ɸ*_1_) that a sequence contains (or does not contain) a BS and the probability distribution *P*_*h *_(*u*_2_|*u*_1_, *ɸ*_2_) for the length of the erroneous shift. In addition, MotifAdjuster estimates the logarithm of the probability that the BS is located on the forward (*v *= 0) or the reverse complementary (*v *= 1) strand, , from the user-provided data as described in subsection *Expectation maximization algorithm*.

The hidden values of *u* lead to the likelihood

(4)

of the data *x* given the model parameters (*λ*, *τ*, *ɸ*), where the sum runs over all possible values of *u*. Here, the subscript *a *stands for *accumulated*, and the subscript *c *stands for *composite*. In the following, we define the likelihood in close analogy to [8,37]. If sequence *x* contains no BS, we assume that *x* is generated by a homogeneous Markov model of order 0; that is,

(5)

If the sequence *x* contains a BS, then *u*_2 _encodes its start position, *u*_3 _encodes its strand, and we assume that the nucleotides upstream and downstream of the BS are generated by a homogeneous Markov model of order 0, yielding

(6)

and

(7)

where the subscript *m *stands for *motif*.

### Prior

As prior of the parameters of the PWM model, we use the "common choice" [34-36] of a product of transformed Dirichlets

(8)

where  denotes the positive hyperparameter of ,  denotes the equivalent sample size (ESS) at position *λ*, which we set to be equal at each position, *α*^*ℓ *^denotes the four-dimensional vector , and *α* denotes the (4 × *w*) matrix (*α*_1_, ..., *α*_*w*_).

The choice of this prior is pragmatic rather than biologically motivated. This prior is conjugate to the likelihood, allowing to write the posterior as a product of transformed Dirichlets. As PWM models are special cases of Bayesian networks, the chosen prior can be understood as a special case of the Bayesian Dirichlet (BD) prior [38].

Analogously, for homogeneous Markov models of order 0, we choose a transformed Dirichlet *P*(*τ*|*β*) := D(*τ*|*β*), where *β*_*a *_denotes the positive hyperparameter of *τ*_*a*_.

MotifAdjuster allows the user to specify *P*(*u*_1_|*ɸ*_1_) and *P*(*u*_2_|*u*_1_, *ɸ*_2_). In principle, MotifAdjuster allows the user to specify any probability distribution *P*(*u*_2_|*u*_1_, *ɸ*_2_) for the length of the erroneous shift, allowing also asymmetric or bimodal distributions, if needed. For an easy and user-friendly execution, MotifAdjuster also offers a discrete and symmetrically truncated Gaussian distribution defined by

(9)

where *z *is an integer value ranging from *-s *to *s*. The real-valued parameter *σ *is similar to the standard deviation of a Gaussian distribution and can be specified by the user, and we denote *ɸ*_2 _:= (*s*, *σ*).

We expect that some sequences are annotated to contain a BS, although they do not contain a BS in reality, but we believe that the fraction of such incorrectly annotated sequences is small. Hence, we choose *P*(*u*_1 _= 0|*ɸ*_1_)=0.2 for the studies presented in this article; that is, we assume that only 20% of the sequences annotated to contain a BS do not contain a BS in reality. We further expect that the annotated position of the BS might be shifted accidentally by a few base pairs, so we choose *s *= 5 and a discrete and symmetrically truncated Gaussian distribution with *σ *= 1. This choice results in a conditional probability of approximately 40% that the BS is not shifted, of approximately 25% that it is shifted 1 bp, and of approximately 5% that it is shifted by more than 1 bp upstream or downstream of the annotated start position, respectively, given that a BS is present in sequence *x*.

As prior of the parameter *ɸ*_3_, we choose a transformed Dirichlet *P*(*ɸ*_3_|*γ*) := *D*(*ɸ*_3_|*γ*) with *γ* = (*γ*_0_, *γ*_1_), where *γ*_*v *_denotes the positive hyperparameter of *ɸ*_3,*v *_with *v *∈ {0, 1}.

Putting all pieces together, we define the prior of the parameters of the mixture model of Equation (4) by:

(10)

stating that we assume *λ*, *τ*, and *ɸ*_3 _to be statistically independent.

We denote the ESS of the mixture model chosen before inspecting any database by *ε*, and we set the ESS of the PWM model to *P*(*u*_1 _= 1|*ɸ*_1_)·*ε*, the positive hyperparameters of the strand parameters to , and the ESS of the homogeneous Markov model of order 0 to (*L* - *P*(*u*_1 _= 1|*ɸ*_1_)·*w*)·*ε*. For the reassessment of BSs presented in this article, we choose an ESS of *ε *= 5, yielding an ESS of 4 for the PWM model, *γ*_0 _= *γ*_1 _= 2, and an ESS of 57 for the homogeneous Markov model of order 0. This choice yields  for every *a* ∈ {*A*, *C*, *G*, *T*} and every *ℓ* ∈ [1, *w*], stating that the chosen prior of the PWM model can be understood as a special case of the BDeu prior [39,40], which in turn is a special case of the BD prior.

### Expectation maximization algorithm

The model parameters of the mixture model defined by Equation (4) cannot be estimated analytically, but any numeric optimization algorithm can be used for maximizing the posterior. One popular optimization algorithm for maximizing the likelihood *P*(S|*λ*, *τ*, *ɸ*) is the EM algorithm [41]. The EM algorithm can be easily modified for maximizing the posterior *P*(*λ*, *τ*, *ɸ*|*S*, *α*, *β*, *γ*) of the data set *S *by iteratively maximizing:

(11)

with

(12)

Q(*λ*, *τ*, *ɸ*, *λ*^(*t*)^, *τ*^(*t*)^|*α*, *β*, *γ*) can be maximized analytically with respect to *λ*, *τ*, and *ɸ*_3_, yielding the familiar expressions provided in Additional data file 2. The posterior *P*(*λ*, *τ*, *ɸ*|*S*, *α*, *β*, *γ*) increases monotonically with each iteration, implying that the modified EM algorithm converges to the global maximum, a local maximum, or a saddle point. We stop the algorithm if the logarithmic increase of the posterior between two subsequent iterations becomes smaller than 10^-6^, restart the algorithm 10 times with randomly chosen initial values of , and choose the parameters of that start with the highest posterior, similar to [8,37]. If we restrict *P*_*h*_(*u*_2_|*u*_1_, *ɸ*_2_) to a uniform distribution over all possible start positions, if we set *P*_*h*_(*u*_3_|*u*_1 _= 1) = 0.5, and if we restrict the background model to be strand symmetric, then we obtain the probabilistic model that is the basis of [8,37].

The flexibility allowed by MotifAdjuster is important for its practical applicability. Typically, the user has prior knowledge about (i) the expected motif occurrence and (ii) the shift distribution, but (iii) no or only limited prior knowledge about the distribution of the BS strand orientation. Hence, we allow the user to specify the logarithm of the probability that a sequence contains a BS *ɸ*_1,0_, a nonuniform distribution to incorporate the prior knowledge of the shift distribution, and we estimate the logarithm of the probability that the BS is located on the forward strand *ɸ*_3,0 _from the data. This setting allows MotifAdjuster to work, without additional intervention, also in the two extreme cases that the BSs lie predominantly either on the forward or on the reverse complementary strand.

Because of the open source license of MotifAdjuster, similar mixture models can be derived and implemented easily, for instance, by using other background and motif models such as Markov models of higher order [42-44], Permuted Markov models [45], Bayesian networks [46,47], or their extensions to variable order [48-53].

## Case studies

In this section we present the results of MotifAdjuster applied to seven data sets of *Escherichia coli*, the validation of MotifAdjuster results for NarL BSs, and the prediction of a novel NarL BS.

### Results for seven data sets of *Escherichia coli*

For testing the efficacy of MotifAdjuster and improving the annotation of BSs of *Escherichia coli*, we extract all data sets with at least 30 BSs of length of at most 25 bp from the bacterial gene-regulatory reference database CoryneRegNet 4.0. The choice of at least 30 BSs of length of at most 25 bp is arbitrary, but motivated by the intention that the results of the following study should not be influenced by TFs with an insufficient number of BSs or by TFs with an atypical BS length. Seven data sets of BSs corresponding to the TFs CpxR, Crp, Fis, Fnr, Fur, Lrp, and NarL satisfy these requirements, and we apply MotifAdjuster to each of these seven data sets. We summarize the results obtained by MotifAdjuster in Table 1, and we provide a complete list of the results in Additional data file 3.

**Table 1:** Annotation results

Gene ID	Gene name	No. BS	BS length	No. removed BSs	No. shifted BSs	Percentage
*b3357*	*crp*	218	22	20	31	23.4%
*b1221*	*narL*	74	7	2	11	17.6%
*b3261*	*fis*	68	21	13	17	44.1%
*b1334*	*fnr*	54	14	2	3	9.3%
*b0683*	*fur*	46	15	1	43	95.7%
*b0889*	*lrp*	43	12	4	23	62.8%
*b3912*	*cpxR*	33	15	9	6	45.5%
Total		536		51	134	34.5%

Summary of the results of the application of MotifAdjuster to all data sets of CoryneRegNet 4.0 from *Escherichia coli *with at least 30 BSs and of at most 25 bp length. Columns 1 and 2 show the gene ID and gene name of the TF; columns 3 and 4 show the number of BSs stored in the database and their lengths; columns 5 and 6 show the number of BSs proposed to be removed and to be shifted; and column 7 shows the percentage of BSs to be removed or shifted. Interestingly, the percentage of proposed adjustments varies strongly from TF to TF, ranging from 9.3% for Fnr to 95.7% for Fur. In summary, we find in the complete data set of 536 BSs that 51 BSs are proposed to be removed and 134 BSs are proposed to be shifted, resulting in 34.5% of the data set being proposed for adjustments.

We find that all of the data sets are considered questionable by MotifAdjuster and, more surprisingly, that 34.5% of the 536 BS annotations are proposed for removal or shifts. The percentage of questionably annotated BSs ranges from 9.3% for Fnr to 95.7% for Fur. MotifAdjuster proposes to remove 51 of the 536 BSs and to shift 134 of the remaining 485 BSs by at least one bp, indicating that, in these seven data sets, erroneous shifts of the annotated BSs are the most frequent annotation error. In particular, the percentage of proposed deletions ranges from 2.2% (one of 46) for Fur to 27.3% (nine of 33) for CprX, and the percentage of proposed shifts ranges from 5.6% (three of 54) for Fnr to 93.5% (43 of 46) for Fur. In more detail, we observe a broad range of shift lengths ranging from one shift 4 bp upstream to two shifts 4 bp downstream, with a sharp peak about 0.

**Figure 1:**
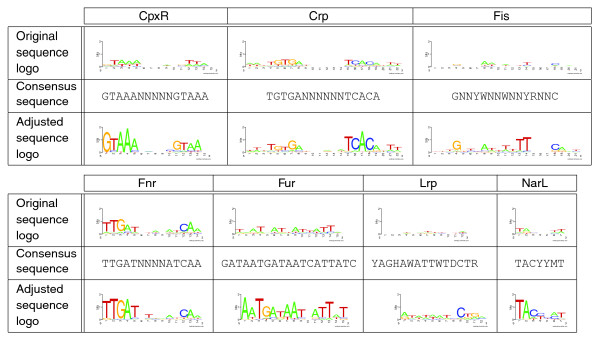
Comparison of binding-site conservation, showing the original sequence logos, the consensus sequences for the TFs obtained from the literature [56-61], and the adjusted sequence logos for the data sets of the TFs CpxR, Crp, Fis, Fnr, Fur, Lrp, and NarL. We find in all seven cases that (i) the adjusted sequence logos show a higher conservation than the original sequence logos, (ii) the adjusted sequence logos are more similar to the consensus sequences than to the original sequence logos; and (iii) clear motifs can be recognized in the adjusted sequence logos of the TFs CpxR, Fur, and NarL that could not be recognized in the original sequence logos.

For each of the seven TFs, we analyze whether the adjustments proposed by MotifAdjuster result in an improved motif of the BSs (Figure 1). We compute the sequence logos [54,55] of the original BSs obtained from CoryneRegNet and those of the BSs proposed by MotifAdjuster, which we call original sequence logos and adjusted sequence logos, respectively. Comparing these sequence logos, we find that the adjusted sequence logos show a higher conservation than the original sequence logos in all seven cases. We also compare the sequence logos with consensus sequences obtained from the literature [56-61], and we find that the adjusted sequence logos are more similar to the consensus sequences than the original sequence logos. In addition, we find, for the TFs CpxR, Fur, and NarL, that the adjusted sequence logos allow us to recognize clear motifs that could not be recognized in the original sequence logos obtained from CoryneRegNet.

We investigate whether there exists any systematic dependence of the observed rate of proposed adjustments exists on the number of BSs, the BS length, and the GC content of the BSs. We find no obvious dependence of the error rate on the number of BSs and on the BS length. Comparing the GC content of the BSs, we find that the GC content of the BSs of all but one TF ranges from 30% to 40%. However, the GC content of the Fur BSs is only 20%. This low GC content might be the reason for the unexpectedly high percentage of shifts in this data set, because it is more likely to shift a BS accidentally in a sequence composed of a virtually binary alphabet.

### Validation of MotifAdjuster results for NarL

To evaluate the previous results, we choose NarL as example and scrutinize the proposed reannotations of MotifAdjuster for this case. The nitrate regulator NarL of *Escherichia coli *is one of the key factors controlling the upregulation of the nitrate respiratory pathway and the downregulation of other respiratory chains. In the absence of oxygen, the energetically most efficient anaerobic respiratory chain uses nitrate and nitrite as electron acceptors [62]. Detection of and adaptation to extracellular nitrate levels are accomplished by complex interactions of a double two-component regulatory system, which consists of the homologous sensory proteins NarQ and NarX, and the homologous TFs NarL and NarP. Depending on the BS arrangement and localization relative to the transcription start site, NarL and NarP act as activators or repressors, thereby enabling a flexible control of the expression of nearly 100 genes.

CoryneRegNet stores 74 NarL BSs, each of length 7 bp (Table 1). Of these 74 BSs, only 36 are considered accurate by MotifAdjuster, whereas 38 are considered to be questionable. In 25 cases, MotifAdjuster proposes to switch the strand orientation of the BS; in five cases, it proposes to shift the location of the BS, and for six BSs, it proposes both a switch of strand orientation and a shift of position. In addition, two BSs are proposed for removal. We present a summary of these results in Table 2, we provide a complete list of the results in Additional data file 4, and we summarize in Table 3 those 13 BSs of the regulator NarL where MotifAdjuster proposes to shift the location of the BS or to remove it from the databases.

**Table 2:** NarL annotation results: Number of binding-site shifts and strand switches

	No strand switch	Strand switch
No position shift	36	25
Position shift	5	6
Removed	2	

Application of MotifAdjuster to the set of 74 NarL BSs results in adjustments proposed for 38 of these BSs. Two BSs are proposed to be removed from the data set. Of the remaining 36 BSs, 25 BSs are labeled with a wrong strand annotation but a correct position, and five BSs are proposed to have a correct strand annotation but a wrong position. For six BSs, both strand annotation and position are proposed to be wrong.

**Table 3:** NarL binding sites with questionable annotations

Gene ID	Gene name	BS	Lit.	Occ.	Shift	Strand	Adj. BS
*b0904*	*focA*	AATAAAT	[63]	1	+1	Reverse	TATTTAT
*b0904*	*focA*	ATAATGC	[63]	1	+1	Forward	TAATGCT
*b0904*	*focA*	ATATCAA	[63]	1	+1	Forward	TATCAAT
*b0904*	*focA*	CAACTCA	[63]	1	+1	Forward	AACTCAT
*b0904*	*focA*	CATTAAT	[63]	1	+1	Reverse	TATTAAT
*b0904*	*focA*	GATCGAT	[63]	1	+1	Reverse	TATCGAT
*b0904*	*focA*	GTAATTA	[63]	1	+1	Forward	TAATTAT
*b0904*	*focA*	TATCGGT	[63]	1	+1	Reverse	TACCGAT
*b0904*	*focA*	TTACTCC	[63]	1	+1	Forward	TACTCCG
*b1223*	*narK*	CACTGTA	[64]	0	-	-	-
*b1224*	*narG*	TAGGAAT	[64]	1	+1	Reverse	AATTCCT
*b4070*	*nrfA*	TGTGGTT	[65]	1	+1	Reverse	TAACCAC
*b4123*	*dcuB*	ATGTTAT	[66]	0	-	-	-

Annotated NarL BSs for which MotifAdjuster proposes either to shift the BS or to remove it from the data set. Columns 1 to 3 contain gene ID, gene name, and the BS (as stored in the database). Column 4 indicates the original literature related to this BS. The following three columns (5 through 7) comprise the three possible adjustments suggested by MotifAdjuster, removal, shift, and strand orientation (relative to the target gene). In column 5, a value of 0 indicates that the BS is proposed for removal, and in column 6, a positive (negative) value denotes a shift of the BS to the right (left). Finally, column 8 provides the adjusted BS. Interestingly, we find that the two BSs that are proposed to be removed are not mentioned in the original literature, and in 10 of the 11 cases, the shifted BS is consistent with the BS published in the original literature. In addition, MotifAdjuster also proposes to switch the BS strand in six of the 11 cases.

To evaluate the accuracy of MotifAdjuster, we check the original literature [63,37] for each of the 13 questionable BS candidates. Comparing both, we find that the proposed annotations agree with those in the literature in all cases but one (BS of gene *b1224*). That is, in 12 of 13 cases signaled by MotifAdjuster as being questionable, the detected error was indeed caused by an inaccurate transfer from the original literature into the gene-regulatory databases RegulonDB and CoryneRegNet. Of those 12 questionable BSs, 10 BSs are correctly proposed to be shifted, and two are correctly proposed to be removed.

Turning to the BS of the gene *b1224*, we find it is published as given in the databases [64], in contrast to the proposal of MotifAdjuster. However, Darwin *et al*. [67] report that a mutation of this BS has little or no effect on the expression of *b1224*. Hence, the proposal could possibly be correct, and the BS could be shifted or even be deleted.

In addition, MotifAdjuster checks the strand annotation of BSs and proposes strand switches if needed. To validate these annotations, we cannot use the annotations from RegulonDB and CoryneRegNet, because these databases contain all BSs in 5'→3' direction relative to the target gene. Hence, we consult annotation experts at the Center for Biotechnology in Bielefeld to reannotate the strand orientation of the BSs manually, and we compare the results with those of MotifAdjuster. Interestingly, we find that the strand orientations proposed by MotifAdjuster are in perfect (100%) agreement with the manually-curated strand orientations. As an independent test of the efficacy of MotifAdjuster for NarL BSs, we use the manually annotated BSs provided by the PRODORIC database [68]. Remarkably, we find also in this case that the results of MotifAdjuster perfectly agree with the annotations.

Another hint that the proposed adjustments of MotifAdjuster could be reasonable is based on the observation that NarL and NarP homodimers bind to a 7-2-7' BS arrangement [61], an inverted repeat structure consisting of a BS on the forward strand, a 2-bp spacer, and a BS on the reverse complementary strand. NarP exclusively binds as homodimer to this 7-2-7' structure. NarL homodimers bind at 7-2-7' sites with high-affinity, but NarL monomers can also bind to a variety of other heptamer arrangements. Instances of this 7-2-7' structure have been reported for four genes: *fdnG*, *napF*, *nirB*, and *nrfA *[61,65]. In contrast to this observation, all BSs in CoryneRegNet as well as RegulonDB are annotated to be on the forward strand, including the second half of the inverted repeat. When applied to these four genes, MotifAdjuster proposes all heptamers of the second half of the 7-2-7' structure to be switched to the reverse strand, in agreement with [61,65]. In addition, MotifAdjuster proposes six additional 7-2-7' BS arrangements, located in the upstream regions of the genes *adhE*, *aspA*, *dcuS*, *frdA*, *hcp*, and *norV*. The positions and the orientations are presented in Additional data file 4.

### Prediction of a novel NarL binding site

After investigating to which degree MotifAdjuster is capable of finding errors in existing gene-regulatory databases, it is interesting to test whether MotifAdjuster could be helpful for finding novel BSs. The flexibility of BS arrangements and the low motif conservation complicate the computational and manual prediction of NarL BSs by curation teams. This results in several cases in which promoter regions are experimentally verified to be bound by NarL, but in which no NarL BS could be detected [69,70]. Examples of such genes are *caiF *[71], *torC *[72], *nikA *[73], *ubiC *[74], and *fdhF *[75]. We extract the upstream regions of these genes, where an upstream sequence is defined by CoryneRegNet as the sequence between positions -560 bp and +20 bp relative to the first position of the annotated start codon of the first gene of the target operon. In addition, we extract those upstream regions of *Escherichia coli *that belong to operons not annotated as being regulated by NarL (background data set).

We investigate whether we can now detect NarL BSs based on the adjusted data set that could not be detected based on the original data set from CoryneRegNet. For that purpose, we estimate the parameters *λ* of the PWM model on the adjusted data set as proposed by MotifAdjuster and *τ* of the homogeneous Markov model on the background data set. From the adjusted PWM, we build a mixture model over both strands with the same probability for each strand; that is, exp(*ɸ*_3,0_) = exp(*ɸ*_3,1_) = 0.5. For the classification of an unknown heptamer *x*, we build a simple likelihood-ratio classifier with these parameters *λ*, *τ*, *ɸ*_3 _and define the log-likelihood ratio by

(13)

For an upstream region, we compute *r*_*max *_defined as the highest log-likelihood ratio of any heptamer *x* in this upstream region. We compute the *P *value of a potential BS *x* with value *r*(*x*) as fraction of the background sequences whose *r*_*max*_-values exceed *r*(*x*).

**Figure 2:**
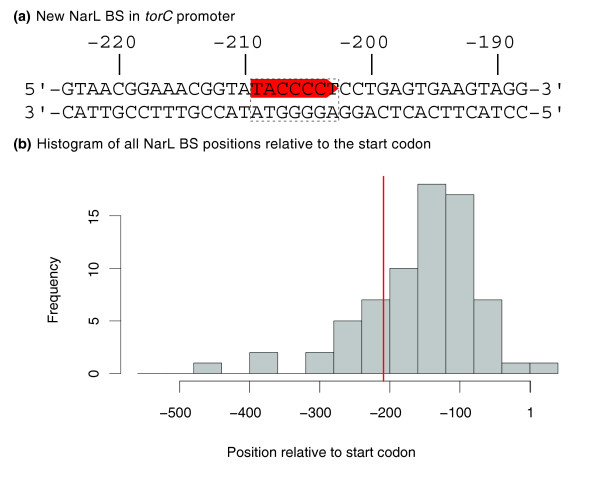
Position of the predicted NarL binding site in the upstream region of *torC*. The NarL BS TACCCT is located on the forward strand with respect to the target operon *torCAD *starting at position -209 bp (red color). All positions are relative to the first nucleotide of the start codon of *torC*. **(a) **The fragment of the upstream region of the *torCAD *operon containing the NarL BS predicted by the PWM model trained on the adjusted data set. **(b) **Histogram of all positions of NarL BSs in the database. The red line indicates the position of the predicted BS.

With this classifier, a significant NarL BS can now be detected in the upstream region of *torC*. Figure 2a shows the double-stranded DNA fragment with the predicted BS (TACCCCT) located on the forward strand starting at -209 bp relative to the start codon, and at -181 bp relative to the annotated transcription start site [76]. The distance of the predicted BS to the start codon agrees with the distance distribution of previously known NarL BS (Figure 2b), providing additional evidence for the predicted BS. This finding closes the gap between sequence-analysis and gene-expression studies, as the *torCAD *operon consists of three genes that are essential for the trimethylamine *N*-oxide (TMAO) respiratory pathway [76]. TMAO is present as an osmoprotector in tissues of invertebrates and can be used as respiratory electron acceptor by *Escherichia coli*. Transcriptional regulation of this operon by NarL binding to the proposed BS would explain nitrate-dependent repression of TMAO-terminal reductase (TorA) activity under anaerobic conditions [72], thereby linking TMAO and nitrate respiration.

## Conclusions

Gene-regulatory databases, such as AGRIS, AthaMap, CoryneRegNet, CTCFBSDB, JASPAR, ORegAnno, PRODORIC, RegulonDB, SCPD, TRANSFAC, TRED, or TRRD store valuable information about gene-regulatory networks, including TFs and their BSs. These BSs are usually manually extracted from the original literature and subsequently stored in databases. The whole pipeline of wet-lab BS identification and annotation, publication, and manual transfer from the scientific literature to data repositories is not just time consuming but also error prone, leading to many false annotations currently present in databases.

MotifAdjuster is a software tool that supports the (re-)annotation process of BSs *in silico*. It can be applied as a quality-assurance tool for monitoring putative errors in existing BS repositories and for assisting with a manual strand annotation. MotifAdjuster maximizes the posterior of the parameters of a simple mixture model by considering the possibilities that (i) a sequence being annotated as containing a BS in reality does not contain a BS; (ii) the annotated BS is erroneously shifted by a few base pairs; and (iii) the annotated BS is erroneously located on the false strand and must be reverse complemented. In contrast to existing *de*-*novo *motif-discovery algorithms, MotifAdjuster allows the user to specify the probability of finding a BS in a sequence and to specify a nonuniform shift distribution.

We apply MotifAdjuster to seven data sets of BSs for the TFs CpxR, Crp, Fis, Fnr, Fur, Lrp, and NarL with a total of 536 BSs, and we find 51 BSs proposed for removal and 134 BSs proposed for shifts. In total, this results in 34.5% of the BSs being proposed for adjustments. We choose NarL as an example to scrutinize the proposed reannotations of MotifAdjuster. Checking the original literature for each of the 13 cases shows that the proposed deletions and shifts of MotifAdjuster are in agreement with the published data. Comparing the strand annotation of MotifAdjuster with independent information indicates that the proposals of MotifAdjuster are in accordance with human expertise. Furthermore, MotifAdjuster enables the detection of a novel BS responsible for the regulation of the *torCAD *operon, finally augmenting experimental evidence of its NarL regulation. MotifAdjuster is an open-source software tool that can be downloaded, extended easily if needed, and used for computational reassessments of BS annotations.

## Availability and requirements

Project name: MotifAdjuster, project home page: [77], operating system(s): platform independent. Programming language: Java 1.5. Requirements: Jstacs 1.2.2. License: GNU General Public License version 3.

## Abbreviations

BS: binding site; EM: expectation maximization; ESS: equivalent sample size; MAP: maximum a posteriori; PWM: position weight matrix; TF: transcription factor.

## Authors' contributions

JK and IG developed the basic idea, and JK implemented MotifAdjuster. JB and TK provided the data. All authors contributed to data analysis, writing, and approved the final manuscript.

## Additional data files

The following additional data are available with the online version of this article. Additional data file 1 contains a comparison of *de-novo *motif-discovery tools including MEME, RecursiveSampler, Improbizer, SeSiMCMC, A-GLAM, and MotifAdjuster for the reannotation of NarL. Additional data file 2 contains a detailed description of the MAP parameter estimators of the model. Additional data file 3 contains a list of MotifAdjuster results for all seven data sets. Additional data file 4 contains a list of MotifAdjuster results compared with the original input of CoryneRegNet and RegulonDB for the TF NarL.

## Supplementary Material

Additional data file 1 Comparison of *de-novo *motif-discovery tools including MEME, RecursiveSampler, Improbizer, SeSiMCMC, A-GLAM, and MotifAdjuster for the reannotation of NarL. Click here for file

Additional data file 2 Detailed description of the MAP parameter estimators of the model. Click here for file

Additional data file 3 List of MotifAdjuster results for all seven data sets. Click here for file

Additional data file 4 List of MotifAdjuster results for the TF NarL compared with the original input of CoryneRegNet and RegulonDB. Click here for file
