# Supplementary material for: MotifAdjuster: a tool for computational reassessment of transcription factor binding site annotations
Source: Genome Biol. 2009 May 1;10(5):R46. doi: 10.1186/gb-2009-10-5-r46 (PMC2718512; doi:10.1186/gb-2009-10-5-r46)
Supplement: Additional data file 1 — Comparison of de-novo motif-discovery tools including MEME, RecursiveSampler, Improbizer, SeSiMCMC, A-GLAM, and MotifAdjuster for the reannotation of NarL. [file gb-2009-10-5-r46-S1.pdf]

# Additional File 1

Recommended by an anonymous reviewer, we present here a comparison of five existing de-novo motif discovery tools and MotifAdjuster when applied to the specific task of re-annotating short BSs. We like to emphasize that such a comparison is intrinsically unfair because none of the five existing tools is specifically tailored to this task, whereas MotifAdjuster is, and the five existing tools are being applied in a very unconventional manner in the following study. Hence, this comparison serves only as a motivation for the development of MotifAdjuster, and it is by no means intended to compare the five selected de-novo motif discovery tools to each other based on this very unconventional application. Table A lists the five selected tools and, for each of them, the chosen parameter values.

We apply the five tools listed in Table A and MotifAdjuster to the NarL data set specified in Table 1. Each of these sequences of length 17 bp contains one annotated NarL BSs of length 7 bp on the forward strand starting at position 6. The re-annotation task is to decide for each sequence (i) if it contains a NarL BS, (ii) at which position the BS starts, and (iii) on which strand the BS is located. Table B shows the annotation from the original literature and the prediction of each of the six tools for 13 sequences for which there is a discrepancy between the database annotation and the original literature.

We find that Improbizer makes only two prediction errors when it fails to predict the BSs of promoters b1223 and b4123 for deletion. The other four of the existing tools propose the correct re-annotation for less than 50% of the BSs. MEME, SeSiMCMC, and A-GLAM propose additional BSs to be shifted or deleted, whereas Improbizer does not propose any other sequence to be re-annotated in addition to those proposed by MotifAdjuster.

| motif discoverer | parameter | value |
|---|---|---|
| MEME | motif distribution | ZOOPS |
| | minimum motif width | 7 |
| | maximum motif width | 7 |
| | maximum number of motifs | 1 |
| RecursiveSampler | Sampler Mode | Recursive Sampler |
| | Number of different motifs | 1 |
| | Max. sites per seq | 1 |
| | Motif Width(s) | 7 |
| | Est. total sites for each motif type | 74 |
| | Fragmentation | FALSE |
| | Prior information | >SBlocks<br>0.2 0.8 |
| | Spacing Model | 1.486720e-06 1.338302e-04 4.431848e-03<br>5.399097e-02 2.419707e-01 3.989423e-01<br>2.419707e-01 5.399097e-02 4.431848e-03<br>1.338302e-04 1.486720e-06 0 0 0 0 0 0 0 |
| Improbizer | Number of motifs to find | 1 |
| | Include Reverse Complement | TRUE |
| | Maximum Occurrences per Sequence | 1 |
| | Initial motif size | 7 |
| | Restrain Expansionist Tendencies | max. value |
| SeSiMCMC | Start motif length | 7 |
| | Adjust motif length | FALSE |
| | Motif absence prior | 0.2 |
| A-GLAM | universial anchor (-4) | 6 |
| | Minimum alignment width (-a) | 7 |
| | Maximum alignment width (-b) | 7 |

Table A: List of five de-novo motif discovery tools and user-specified parameters. The table shows for each tool only those parameters that deviate from the default values. The names of the tools can be used as links to the corresponding web sites.

| gene ID | annotated site | literature | MEME | RecursiveSampler | Improbizer | SeSiMCMC | A-GLAM | MotifAdjuster |
|---|---|---|---|---|---|---|---|---|
| b0904 | aataaat | 1 | - | -1 | 1 | -2 | 2 | 1 |
| b0904 | ataatgc | 1 | 1 | 1 | 1 | - | 1 | 1 |
| b0904 | atatcaa | 1 | - | 1 | 1 | 0 | - | 1 |
| b0904 | caactca | 1 | - | -2 | 1 | - | 1 | 1 |
| b0904 | cattaat | 1 | - | -2 | 1 | -1 | - | 1 |
| b0904 | gatcgat | 1 | - | 1 | 1 | - | - | 1 |
| b0904 | gtaatta | 1 | 1 | 0 | 1 | 0 | 1 | 1 |
| b0904 | tatcggt | 1 | 1 | 1 | 1 | -2 | -4 | 1 |
| b0904 | ttactcc | 1 | 1 | 1 | 1 | 0 | 1 | 1 |
| b1223 | cactgta | - | 5 | -1 | 0 | 4 | - | - |
| b1224 | taggaat | 1 | - | -2 | 1 | 0 | 0 | 1 |
| b4070 | tgtggtt | 1 | 1 | 1 | 1 | 2 | - | 1 |
| b4123 | atgttat | - | - | -1 | -1 | 0 | -5 | - |
| correct | | | 5 | 6 | 11 | 0 | 4 | 13 |
| Further Predicted Deletions | | | 15 | 0 | 0 | 5 | 15 | 0 |
| Further Predicted Shifts | | | 6 | 3 | 0 | 56 | 18 | 0 |

Table B: Predictions of BSs in the NarL data set. The symbol "-" indicates that the corresponding de-novo motif discovery tool proposes this BS for deletion, and any number $v$ indicates that it proposes to shift the BS by $v$ bp downstream.
