# Supplementary material for: MotifAdjuster: a tool for computational reassessment of transcription factor binding site annotations
Source: Genome Biol. 2009 May 1;10(5):R46. doi: 10.1186/gb-2009-10-5-r46 (PMC2718512; doi:10.1186/gb-2009-10-5-r46)
Supplement: Additional data file 2 — Detailed description of the MAP parameter estimators of the model. [file gb-2009-10-5-r46-S2.pdf]

Additional File 2

The function $Q(\underline{\lambda}, \underline{\tau}, \underline{\phi}, \underline{\lambda}^{(t)}, \underline{\tau}^{(t)}, \underline{\phi}^{(t)} | \underline{\alpha}, \underline{\beta}, \underline{\gamma})$ from Equation 11 can be maximized analytically with respect to $\underline{\lambda}, \underline{\tau}$, and $\underline{\phi}_3$, yielding the familiar expressions

$$\exp\left(\lambda_a^{\ell\,(t+1)}\right) = \frac{H_a^\ell + \alpha_a^\ell}{H_\cdot^\ell + \alpha_\cdot^\ell} \tag{1a}$$

$$\exp\left(\tau_a^{\,(t+1)}\right) = \frac{H_a + G_a + \beta_a}{H_\cdot + G_\cdot + \beta_\cdot} \tag{1b}$$

$$\exp\left(\phi_{3,v}^{(t+1)}\right) = \frac{F_v + \gamma_v}{F_\cdot + \gamma_\cdot} \tag{1c}$$

for $v \in \{0,1\}, \ell \in [1,L], a \in \{A,C,G,T\}$, and

$$w_0^{(t)}(\underline{x}) := \frac{P_b(\underline{x}|\underline{\tau}^{(t)}) P_h(u_1 = 0|\underline{\phi_1})}{P_a(\underline{x}|\underline{\lambda}^{(t)}, \underline{\tau}^{(t)}, \underline{\phi}^{(t)})} \tag{2a}$$

$$w_0^{(t)} := \sum_{\underline{x}\in S} w_0^{(t)}(\underline{x}) \tag{2b}$$

$$w_1^{(t)} := \sum_{\underline{x}\in S} \sum_{u_2=-s}^{s} \sum_{u_3} w_{1,u_2,u_3}^{(t)}(\underline{x}) \tag{2c}$$

$$H_a^\ell := \sum_{\underline{x}\in S} \sum_{u_2=-s}^{s} w_{1,u_2,0}^{(t)}(\underline{x}) \delta_{x_{s+u_2+\ell},a} + w_{1,u_2,1}^{(t)}(\underline{x}) \delta_{x^{RC}_{s+u_2+w+1-\ell},a} \tag{2d}$$

$$H_a := \sum_{\underline{x}\in S} \sum_{u_2=-s}^{s} w_{1,u_2,1}^{(t)}(\underline{x}) \cdot \left(\sum_{\ell=1}^{s+u_2} \delta_{x_\ell,a} + \sum_{\ell=s+u_2+w+1}^{L} \delta_{x_\ell,a} \right) \tag{2e}$$

$$G_a := \sum_{\underline{x}\in S} w_0^{(t)}(\underline{x}) \cdot \sum_{\ell=1}^{L} \delta_{x_\ell,a} \tag{2f}$$

$$F_v := \sum_{\underline{x}\in S} \sum_{u_2=-s}^{s} w_{1,u_2,v}^{(t)}(\underline{x}) \tag{2g}$$

where δ denotes the Kronecker symbol, which is equal to 1 if both indices are identical, and equal to 0 otherwise. H_a^ℓ denotes the weighted absolute frequency of finding nucleotide a at position ℓ in the set of BSs, H_a denotes the weighted absolute frequency of finding nucleotide a upstream or downstream of the BSs, G_a denotes the weighted absolute frequency of finding nucleotide a in sequences that do not contain a BS, and finally F_0 (F_1) denotes the weighted absolute frequency of finding a BS on the forward (reverse complementary) strand. The dotted variables denote the sums over the corresponding index of these variables, e.g. $H_\cdot^\ell := \sum_a H_a^\ell$ or $\alpha_\cdot^\ell := \sum_a \alpha_a^\ell$.
